# PaO_2_/FiO_2_ and SpO_2_/FiO_2_ ratios revisited: Useful, misleading, or both?

**DOI:** 10.1016/j.jointm.2026.04.001

**Published:** 2026-05-17

**Authors:** Prashant Nasa, Denise Battaglini, Deven Juneja, Marcus J. Schultz, Jozef Kesecioglu

**Affiliations:** 1 Department of Anaesthesia and Critical Care Medicine, The Royal Wolverhampton Trust, New Cross Hospital, Wolverhampton, United Kingdom; 2 Department of Intensive Care Medicine, Amsterdam University Medical Centre, Amsterdam, the Netherlands; 3 Anesthesia and Intensive Care, IRCCS Ospedale Policlinico San Martino, Genoa, Italy; 4 Department of Surgical Sciences and Integrated Diagnostics, University of Genoa, Genoa, Italy; 5 Institute of Critical Care Medicine, Max Super Speciality Hospital, New Delhi, India; 6 Mahidol–Oxford Tropical Medicine Research Unit (MORU), Mahidol University, Bangkok, Thailand; 7 Nuffield Department of Medicine, University of Oxford, Oxford, United Kingdom; 8 Department of Anaesthesiology, General Intensive Care and Pain Medicine, Division of Cardiac Thoracic Vascular Anesthesia and Intensive Care Medicine, Medical University Vienna, Währinger Gürtel, Vienna, Austria; 9 Department of Intensive Care Medicine, University Medical Center Utrecht, Utrecht University, Utrecht, the Netherlands; 10 Department of Anaesthesiology, Rescue- and Pain Medicine, Cantonal Hospital St. Gallen, HOCH Health Ostschweiz, St. Gallen, Switzerland

**Keywords:** Acute respiratory distress syndrome, Partial pressure of oxygen to fraction of inspired oxygen (PaO_2_/FiO_2_), Peripheral oxygen saturation to fraction of inspired oxygen (SpO_2_/FiO_2_), Oxygenation ratios, Pulse oximetry, Acute hypoxemic respiratory failure

## Abstract

The ratio of arterial partial pressure of oxygen to the inspired oxygen fraction (PaO_2_/FiO_2_) serves as a cornerstone metric for the management of patients with acute respiratory distress syndrome (ARDS). This ratio is integral to the Berlin definition of ARDS and is used for severity classification, prognostication, and guiding therapeutic interventions. Furthermore, the PaO_2_/FiO_2_ ratio has been employed in landmark trials to ascertain patients’ eligibility for interventions such as prone positioning and extracorporeal membrane oxygenation. The modifications of the Berlin definition, the Kigali modification, and the 2023 New Global definition suggested using the ratio of peripheral oxygen saturation to FiO_2_ (SpO_2_/FiO_2_) as a surrogate for the PaO_2_/FiO_2_ ratio when arterial blood gas is inaccessible. Although these oxygenation ratios are valuable for timely recognition and categorizing ARDS, as well as informing clinical decision-making, several significant limitations limit their efficacy, such as dependence on ventilator settings and FiO_2_. The isolated use of these oxygenation ratios may not accurately monitor the evolution of the pathology and response to interventions. Additionally, the arbitrary cutoffs established for these oxygenation metrics frequently result in overlapping categories rather than distinct subgroups of ARDS. Finally, pulse oximetry measurements can be compromised by factors such as patient motion, perfusion variability, dyshemoglobinemia, and skin pigmentation-related bias, which may obscure hypoxemia. Considering these challenges, recent research has explored the trajectory of the PaO_2_/FiO_2_ ratio relative to static measurements and the reclassification of ARDS severity after 24–48 h for prognostication. Alternative metrics such as the oxygenation index, oxygenation saturation index, and (PaO_2_ ×10)/(FiO_2_ × PEEP) ratio (which incorporates positive end-expiratory pressure) have demonstrated greater ability to predict patients’ outcomes. This review summarizes the evidence base, clinical applications, and pitfalls of PaO_2_/FiO_2_ and SpO_2_/FiO_2_ ratios in ARDS and potential alternatives, particularly in the context of diagnosis, categorization, and management of ARDS.

## Introduction

Assessment of gas exchange and hypoxemia remains a cornerstone of managing critically ill and perioperative patients. For decades, the oxygenation ratio, described as the partial pressure of oxygen (PaO_2_) to the fraction of inspired oxygen (FiO_2_) (PaO_2_/FiO_2_ ratio), has guided clinical decisions, diagnosis, severity classification, research categorization, and guideline development.^[^^1^^]^ Originally conceived for stratification of acute respiratory distress syndrome (ARDS), it is now widely used for treatment escalation, resource allocation, and prognostication of other forms of respiratory failure, far beyond its original scope.^[^^2^^]^ Yet mounting evidence questions this reliance.

Another oxygenation ratio in which oxygen saturation from a pulse oximeter (SpO_2_) replaces PaO_2_, SpO_2_/FiO_2_ ratio, has emerged as a noninvasive and feasible alternative, especially in resource-limited settings.^[^^3^^]^ The indices such as PaO_2_/FiO_2_ and SpO_2_/FiO_2_ ratios offer a simple, widely applicable, and clinically useful approach for rapid severity stratification and guiding therapeutic interventions, including in nonspecialist settings. However, oxygenation composite metrics incorporating lung mechanics and gas exchange offer potentially superior insights. Meanwhile, advances in monitoring, such as lung ultrasound and electrical impedance tomography, challenge the assumption that a single-point oxygenation measurements adequately capture the complexity of respiratory failure.^[^^4^^]^

This review explores the evolution, evidence, and limitations of oxygenation ratios and delves into the emerging oxygenation metrics. As healthcare systems increasingly prioritize resource efficiency and precision-based interventions, the time has come to assess whether our traditional approaches to oxygenation assessment still serve modern clinical practice, or whether a shift toward more comprehensive, precise strategies is needed to optimize patient management and outcomes.

## History of Oxygenation Ratios

The first description of ARDS provided by David Ashbaugh in 1967^[^^5^^]^ emphasized the hypoxia that was refractory to conventional oxygen therapy. In 1988, Murray et al.^[^^6^^]^ proposed a Lung Injury Score as an expanded definition that incorporated key pathophysiological features of ARDS. This framework incorporated the PaO_2_/FiO_2_ ratio as a component reflecting the severity of lung injury, alongside other parameters such as positive end-expiratory pressure (PEEP), respiratory system compliance, and chest radiographic findings. Furthermore, while a PEEP was included in the Lung Injury Score, it is important to note that the PaO_2_/FiO_2_ ratio was scored independently of the PEEP level_._ The American-European Consensus Conference delineated acute lung injury and ARDS using PaO_2_/FiO_2_ ratios of ≤300 and <200 mmHg, respectively.^[^^7^^]^ Notably, this definition also failed to specify ventilator settings, particularly minimum PEEP and FiO_2_ levels, at the time of measurement. Ferguson et al.,^[^^8^^]^ proposed a minimum PEEP of 10 cmH_2_O for the assessment of oxygenation in the first Delphi definition.

Villar et al.,^[^^9^^]^ prospectively evaluated different combinations of PEEP and FiO_2_ settings in a cohort of patients with ARDS and demonstrated that ventilator settings of PEEP ≥ 10 cmH_2_O and FiO_2_ ≥ 0.5 on day 1, were associated with significant difference in intensive care unit (ICU) mortality across categories of acute lung injury, ARDS, and acute hypoxemic respiratory failure (AHRF).^[^^9^^]^ In a separate multicenter prospective study, the severity categorization of ARDS based on PaO_2_/FiO_2_ measurements with a PEEP ≥ 10 cmH_2_O and FiO_2_ ≥ 0.5 on day 1 was associated with significant differences in mortality across categories.^[^^10^^]^

Subsequently, the Berlin definition reduced the minimum level of PEEP to 5 cmH_2_O for PaO_2_/FiO_2_ ratio measurement on the basis of feasibility.^[^^11^^]^ Additionally, the PaO_2_/FiO_2_ ratio was used to stratify ARDS into mild (201 to ≤300 mmHg), moderate (101 to ≤200 mmHg), and severe (≤100 mmHg) categories. The thresholds for the PaO_2_/FiO_2_ ratio in the New Global definition remain unchanged.^[^^12^^]^

The SpO_2_/FiO_2_ ratio was proposed by Rice et al.,^[^^13^^]^ as an alternative oxygenation ratio for the diagnosis and follow-up of ARDS patients in situations where arterial blood gas (ABG) is unavailable.^[^^13^^]^ The Kigali modification of the Berlin definition of ARDS has also reported a comparative efficacy of the SpO_2_/FiO_2_ ratio relative to the PaO_2_/FiO_2_ ratio in diagnosing ARDS, based on a single-center study, conducted in a resource-constrained setting of Rwanda.^[^^14^^]^ The new Global definition incorporated SpO_2_/FiO_2_ as an alternative to PaO_2_/FiO_2_ ratio in resource-limited settings, for the diagnosis of hypoxia and for classification of severity of ARDS, provided the SpO_2_ is ≤97%.^[^^10^^]^

## Evidence on Oxygenation Ratios

The PaO_2_/FiO_2_ ratio for defining hypoxemia in ARDS was formally incorporated into the American-European Consensus Conference definition through expert consensus.^[^^7^^]^ The Berlin definition used a Delphi-based consensus process involving an international panel to draft a definition and to propose a PaO_2_/FiO_2_ ratio-based categorization of ARDS. The definition and severity categories were validated through a meta-analysis of patient-level datasets from four multicentric clinical trials involving over 4000 patients. The mortality rates, median duration of ventilation, and ventilator-free days were inversely proportional to the PaO_2_/FiO_2_ ratios. The thresholds for PaO_2_/FiO_2_ ratios were merely developed through consensus for practical considerations in grading outcomes, including mortality, along a continuum, rather than relying on strict physiological inflection points. Therefore, it may seem reasonable to think that the PaO_2_/FiO_2_ thresholds are predictive of mortality in ARDS. However, the area under the receiver operating characteristic curve (AUROC) for the Berlin definition was only 0.577 (95% confidence intervals [CI]: 0.561 to 0.593).^[^^11^^]^ Moreover, the Berlin definition does not clearly specify the optimal timing for severity assessment using PaO_2_/FiO_2_ ratios (e.g., at admission, within the first 24 h or at the worst point within the first 48 h), nor the extent of ventilator optimization before measurement. This lack of standardization may influence the classification of ARDS severity, as oxygenation indices can vary considerably depending on ventilator settings and the timing of assessment.^[^^9^^,^^10^^]^

A few clinical trials and guidelines have attempted to categorize ARDS using a single PaO_2_/FiO_2_ cutoff of 150 mmHg, distinguishing mild-moderate from moderate-severe disease.^[^[15], [16], [17]^]^ Notably, several landmark ARDS trials, including those evaluating neuromuscular blockade and prone positioning***,*** have used a PaO_2_/FiO_2_ threshold of 150 mmHg for patient selection.^[^^15^^,^^16^^]^ Moderate-severe ARDS (PaO_2_/FiO_2_ <150 mmHg) had worse physiological and anatomical derangements (such as peak pressure, arterial partial pressure of carbon dioxide [PaCO_2_], pH, heavier lungs, and greater inhomogeneity), greater lung recruitability and higher mortality risk compared with mild-moderate ARDS (PaO_2_/FiO_2_ 150 to ≤300 mmHg).^[^^18^^]^ Yet, the accuracy of PaO_2_/FiO_2_ <150 mmHg for predicting mortality was low with an AUROC of 0.56 (95%CI: 0.51 to 0.61).^[^^19^^]^

The SpO_2_/FiO_2_ ratio derived from pulse oximetry possesses inherent advantages such as non-invasiveness, continuous, ease of availability, and affordability and is a fair surrogate of PaO_2_, with SpO_2_/FiO_2_ ratios of 315 and 235 corresponding to PaO_2_/FiO_2_ ratios of 300 mmHg (91% sensitivity, 56% specificity) and 200 mmHg (86% sensitivity, 85% specificity).^[^^20^^]^ However, the relationship between SpO_2_ and PaO_2_ is nonlinear and becomes inaccurate above 96% because of the flat portion of oxyhemoglobin curve.^[^^13^^]^ In a large mixed cohort with AHRF including pneumonia, Rice et al.,^[^^13^^]^ found that SpO_2_/FiO_2_=235 corresponded to PaO_2_/FiO_2_=200, and SpO_2_/FiO_2_=315 to PaO_2_/FiO_2_=300, The study reported a strong correlation between SpO_2_/FiO_2_ ratio and PaO_2_/FiO_2_ ratio, demonstrating a linear relationship described by the equation^[^^13^^]^:

$$\;\;\;\;\;\text{Sp}O_{2}/\text{Fi}O_{2}=64+0.84(\text{Pa}O_{2}/\text{Fi}O_{2})$$

However, the misclassification of ARDS severity persisted in about 30% of cases. The authors emphasized that FiO_2_ standardization is critical for reliable interpretation.^[^^13^^]^ The correlation was further supported by a secondary analysis of the National Heart, Lung, and Blood Institute ARDS Network trials.^[^[21], [22], [23]^]^ Additionally, secondary analysis of prospective observational study on COVID-19-related ARDS indicated a good correlation between SpO_2_/FiO_2_ or PaO_2_/FiO_2_ ratios in predicting patient outcomes.^[^^24^^]^ Moreover, imputed SpO_2_/FiO_2_ values corresponding to PaO_2_/FiO_2_ ratios have also been validated within the *Sequential Organ Failure Assessment (SOFA) score* and have been shown to predict clinical outcomes^[^^25^^]^ (Table 1).

**Table 1:** Adult studies showing prediction of PaO_2_/FiO_2_ from SpO_2_/FiO_2_ ratios.

Authors	Patient population	Sample size	Correlation between SpO_2_/FiO_2_ and PaO_2_/FiO_2_	Statistical results
Rice et al.^[^^13^^]^	ARDS patients under MV	672 for derivation and 402 for validation	SpO_2_/FiO_2_=235 indicated PaO_2_/FiO_2_=200 SpO_2_/FiO_2_=315 indicated PaO_2_/FiO_2_=300	NA
Tripathi et al.^[^^26^^]^	Patients on IMV as a part of general anesthesia	2754	SpO_2_/FiO_2_=206 indicated PaO_2_/FiO_2_=300 SpO_2_/FiO_2_=180 indicated PaO_2_/FiO_2_=200	NA
Namendys-Silva et al.^[^^27^^]^	All ICU patients	232	SpO_2_/FiO_2_ ≤ 512 indicating PaO_2_/FiO_2_ ≤ 400 SpO_2_/FiO_2_ ≤ 357 indicating PaO_2_/FiO_2_ ≤ 300 SpO_2_/FiO_2_ ≤ 214 indicating PaO_2_/FiO_2_ ≤ 200 SpO_2_/FiO_2_ ≤ 89 indicating PaO_2_/FiO_2_ ≤ 100	NA
Serpa Neto et al.^[^^28^^]^	Patients with sepsis	260	SpO_2_/FiO_2_=154 indicated PaO_2_/FiO_2_=100 SaO_2_/FiO_2_=241 indicated PaO_2_/FiO_2_=300	NA
Schmidt et al.^[^^29^^]^	All patients under MV	3767	SpO_2_/FiO_2_=295 indicated PaO_2_/FiO_2_ ≤ 300	99% sensitivity and 9.9% specificity
Bass et al.^[^^30^^]^	Patients with ARF under MV	77	SpO_2_/FiO_2_ ≤ 315 indicated PaO_2_/FiO_2_ ≤ 300 SpO_2_/FiO_2_ ≤ 235 indicated PaO_2_/FiO_2_ ≤ 200	Sensitivity of 83% and specificity of 50% Sensitivity of 70% and specificity of 90%
Bashar et al.^[^^31^^]^	ICU post-operative after CABG	175 for derivation and 358 for validation	SpO_2_/FiO_2_=311 indicated PaO_2_/FiO_2_=300	Sensitivity of 90% and specificity of 80%
Babu et al.^[^^32^^]^	Respiratory failure admitted in surgical ICU	300	SpO_2_/FiO_2_=285 indicated PaO_2_/FiO_2_=200 SpO_2_/FiO_2_=323 indicated PaO_2_/FiO_2_=300	Sensitivity of 73% and specificity of 77% Sensitivity of 72% and specificity of 73%
Kumar et al.^[^^33^^]^	ARF admitted in COVID ICU	80	SpO_2_/FiO_2_=219 indicated PaO_2_/FiO_2_=200 SpO_2_/FiO_2_=299 indicated PaO_2_/FiO_2_=300	NA
Gandhi et al.^[^^34^^]^	Respiratory failure admitted in COVID ICU	100	SpO_2_/FiO_2_=205 indicated PaO_2_/FiO_2_=200 SpO_2_/FiO_2_=290 indicated PaO_2_/FiO_2_=300	NA
Choudhury et al.^[^^35^^]^	COVID-19 patients admitted to ICU	396	SpO_2_/FiO_2_=395 indicated PaO_2_/FiO_2_=100 SpO_2_/FiO_2_=405 indicated PaO_2_/FiO_2_=200 SpO_2_/FiO_2_=411 indicated PaO_2_/FiO_2_=300	Sensitivity of 66.7% and specificity of 73.5% Sensitivity of 71.8% and specificity of 70.8% Sensitivity of 65% and specificity of 80%
Alur et al.^[^^36^^]^	ICU patients with AHRF	125	SpO_2_/FiO_2_=252 indicated PaO_2_/FiO_2_=200 SpO_2_/FiO_2_=321 indicated PaO_2_/FiO_2_=300	Sensitivity of 69% and specificity of 95%

AHRF, Acute hypoxic respiratory failure; ARDS, Acute respiratory distress syndrome; ARF, Acute respiratory failure; CABG, Coronary artery bypass grafting; ICU, Intensive care unit; MV, Mechanical ventilation; PaO_2_/FiO_2_, Partial pressure of arterial oxygen by fraction of inspired oxygen; SpO_2_/FiO_2_, Oxygen saturation by fraction of inspired oxygen.

## Oxygenation Ratios Beyond ARDS

The PaO_2_/FiO_2_ ratio serves as a gold standard for assessing the severity of hypoxemia for ARDS. However, clinicians increasingly use the PaO_2_/FiO_2_ ratio and ARDS severity thresholds (e.g., PaO_2_/FiO_2_ ≤300 or ≤200 mmHg) in other contexts such as pneumonia, sepsis, perioperative care, and determining the need for respiratory supports like high-flow nasal oxygen (HFNO), noninvasive ventilation (NIV). Moreover, the severity of hypoxemia has been shown to be an independent predictor of mortality in critically ill patients, regardless of the underlying etiology.^[^^37^^]^ Furthermore, SpO_2_/FiO_2_ ratio is used as an alternative to PaO_2_/FiO_2_ ratio in many of these conditions, where ABG analysis access is limited.

### Pneumonia

The PaO_2_/FiO_2_ ratio has been used in patients with community-acquired pneumonia (CAP) to determine the severity of hypoxemia, to assess the need for respiratory support, and to predict the outcomes.^[^^38^^]^ Clinical risk scores such as SMART-COP, which includes the PaO_2_/FiO_2_ ratio, are valuable in predicting the severity of CAP and the need for respiratory or vasopressor support.^[^^39^^]^ The oxygenation indices can also support clinical decision-making by helping to guide the escalation of respiratory support and monitor response to therapy. Furthermore, worsening hypoxemia, as reflected by declining PaO_2_/FiO_2_ ratios, may identify patients at increased risk of progression to ARDS, particularly when invasive ventilation becomes necessary.^[^^40^^]^ Collectively, these observations highlight the importance of oxygenation indices not only in severity assessment but also in anticipating disease trajectory in pneumonia.

The noninvasive nature of SpO_2_/FiO_2_ made it valuable, especially where ABGs were limited. In adult pneumonia patients, both COVID-19 and non-COVID-19 related, contemporaneous SpO_2_/FiO_2_ and PaO_2_/FiO_2_ ratios correlate moderately to strongly, but clinically relevant dispersion limits their interchangeability.^[^^33^^,^^41^^]^ However, wide prediction intervals shown in other settings may reduce their standalone reliability. One multicenter study of patients with COVID-19 pneumonia across wards and ICUs demonstrated that SpO_2_/FiO_2_ could be used as a surrogate of PaO_2_/FiO_2_ ratio in patients and could be highly useful in resource-limited settings during this COVID-19 pandemic. However, the initial SpO_2_/FiO_2_ ratio on admission could not indicate the need for invasive ventilation.^[^^42^^]^

In pneumonia (COVID-19 and non-COVID-19), SpO_2_/FiO_2_ provides a practical, noninvasive screening and trending tool. It correlates with PaO_2_/FiO_2_ but with a risk of clinically relevant misclassification at decision thresholds. For research eligibility, prognosis, or ventilator management, the PaO_2_/FiO_2_ ratio is preferable due to greater precision. For triage and early recognition of deterioration, the SpO_2_/FiO_2_ ratio is acceptable, with the caveat that SpO_2_ >97% blunts its reliability due to a plateau in the oxyhemoglobin curve.

### Sepsis and septic shock

The PaO_2_/FiO_2_ ratio is important for the diagnosis of sepsis as a variable of the SOFA score. The cutoffs of the PaO_2_/FiO_2_ ratio used in SOFA were also similar to those for severity grading of ARDS, with a higher score for a low PaO_2_/FiO_2_. However, the thresholds for the PaO_2_/FiO_2_ ratio were changed in the SOFA-2 score (Table 2). Although, the accuracy of predicting mortality for overall SOFA–2 (AUROC 0.79, 95%CI: 0.76 to 0.81) was similar to SOFA (AUROC 0.77, 95%CI: 0.74 to 0.81), the revised cutoffs using 10 kPa (75 mmHg) intervals for PaO_2_/FiO_2_ ratio, outperformed the respiratory SOFA score (AUROC 0.615, 95%CI: 0.607 to 0.622 *vs.* AUROC 0.610, 95%CI: 0.603 to 0.618).^[^[43], [44], [45]^]^ Additionally, lower PaO_2_/FiO_2_ (<300 mmHg) ratios have been shown to be independently associated with increased mortality in patients with sepsis.^[^^46^^]^ In a retrospective analysis of MIMIC-IV database, the relationship between PaO_2_/FiO_2_ ratio and mortality was U-shaped and PaO_2_/FiO_2_ ratios between 183 and 219 mmHg were found to be associated with a lower risk of 28-day mortality.^[^^47^^]^

**Table 2:** Comparison of respiratory scoring criteria (PaO_2_/FiO_2_) between SOFA and SOFA‑2.

Score	SOFA	SOFA-2
0	≥400 mmHg (53.3 kPa)	>300 mmHg (>40.0 kPa) or SpO_2_/FiO_2_ >300 mmHg
1	<400 (53.3)	≤300 mmHg (≤40.0 kPa) or SpO_2_/FiO_2_ ≤300 mmHg
2	<300 mmHg (40.0 kPa)	≤225 mmHg (≤30.0 kPa) or SpO_2_/FiO_2_ ≤250 mmHg
3	<200 mmHg (26.7 kPa) with respiratory support	≤150 mmHg (≤20.0 kPa) and advanced ventilator support or SpO_2_/FiO_2_ ≤200 mmHg
4	<100 mmHg (13.3 kPa) with respiratory support	≤75 mmHg (≤10.0 kPa) and advanced ventilator support or ECMO or SpO_2_/FiO_2_ ≤120 mmHg

ECMO, Extracorporeal membrane oxygenation; SaO_2_/FiO_2_, Oxygen saturation by fraction of inspired oxygen; SOFA, Sepsis-related organ failure assessment.

SOFA-2 score also incorporates corresponding SpO_2_/FiO_2_ ratios when ABG is unavailable. Peripheral perfusion abnormalities associated with worsening shock, due to vasoconstriction or low pulsatility, could be a major concern. Although a low SpO_2_/FiO_2_ ratio at ICU admission in patients with sepsis and septic shock was associated with increased mortality risk, especially the lower values; the correlation with the PaO_2_/FiO_2_ ratio was only moderate (*r* = 0.48).^[^^28^^]^ In another study in the emergency department, pulse oximetry overestimated arterial oxygen saturation (SaO_2_) measured by ABG in patients with severe sepsis or septic shock, with larger errors at lower saturations.^[^^48^^]^ Hence, in sepsis, PaO_2_/FiO_2_ is more reliable when obtainable. If SpO_2_/FiO_2_ has to be used as a noninvasive alternative, SpO_2_ should be kept <97%, and near decision thresholds should be interpreted cautiously.^[^^49^^]^

### Pulmonary embolism

The PaO_2_/FiO_2_ ratio has been used for risk stratification in patients with pulmonary embolism and for identifying patients with a higher risk of deterioration.^[^^50^^]^ The PaO_2_/FiO_2_ ratio <265 mmHg has been identified as an increased risk of in-hospital death, though other studies suggest slightly different cutoffs.^[^^51^^]^ Furthermore, the PaO_2_/FiO_2_ ratio has been used to estimate the severity of pulmonary artery obstruction, as it is negatively correlated with the pulmonary artery computed tomography obstruction index. A lower PaO_2_/FiO_2_ ratio corresponds to a more severe obstruction.^[^^52^^]^ Although the PaO_2_/FiO_2_ ratio may correlate with severity in pulmonary embolism, oxygenation parameters alone do not play a pivotal role in therapeutic decision-making, which is primarily guided by hemodynamic status, right ventricular function, and cardiac biomarkers.

### Perioperative and anesthesia settings

In the operating room and postanesthetic care unit, FiO_2_ delivery fluctuates rapidly, and lung mechanics change with anesthesia and recruitment. Intra or postoperative oxygenation is confounded by high FiO₂, variable PEEP, atelectasis, and recruitment manoeuvres—factors that can acutely swing PaO_2_/FiO_2_ without reflecting intrinsic lung pathology. These factors limit the reliability of both PaO_2_/FiO_2_ and SpO_2_/FiO_2_ thresholds derived from resting or ventilated ARDS populations. Although perioperative studies exist for PaO_2_/FiO_2_ ratio,^[^^53^^]^ or comparing oxygen reserve index *vs*. SpO₂ for hypoxemia detection, they generally support pulse oximetry for trend monitoring, but not for applying ARDS–based cutoffs.^[^^54^^]^

## Oxygenation Ratios with Respiratory Support

Respiratory support modalities, such as HFNO, continuous positive airway pressure, bilevel positive airway pressure, NIV, invasive mechanical ventilation, or long–term home ventilation, are often used in the management of respiratory failure. The use of PaO_2_/FiO_2_ and SpO_2_/FiO_2_ ratios with these modalities is influenced by the settings and PEEP levels.

Estimation of FiO_2_ during conventional oxygen therapy can be challenging, particularly when oxygen is delivered via standard face masks or nasal canula. FiO_2_ is commonly estimated using conversion tables based on oxygen flow rates. However, the actual delivered FiO_2_ can vary considerably when estimated by these equations, depending on factors such as the patient’s inspiratory flow, breathing pattern, and mask fit, and consequently exaggerate the severity of hypoxemia, calculated by PaO_2_/FiO_2_ or SpO_2_/FiO_2_ ratios.^[^^55^^]^ This methodological limitation should be considered when interpreting studies comparing different noninvasive respiratory support strategies.

### High-flow nasal oxygen

In HFNO, FiO_2_ delivery is more stable at higher flows but still affected by mouth opening, patient inspiratory demand, respiratory rate, and nasal prong fit. Moreover, the physiological benefits of HFNO are due to flow-dependent positive nasopharyngeal pressure, expiratory resistance (PEEP), thereby reducing respiratory rate and effort. However, the benefits are dependent on the patients’ ability to close their mouths while on HFNO.^[^[56], [57], [58]^]^ Thus variable FiO2 and PEEP delivery may perturb both PaO_2_/FiO_2_ and SpO_2_/FiO_2_ ratios, independently of underlying disease severity. This leads to greater heterogeneity and overestimation of ARDS severity.^[^^59^^]^ Consequently, PaO_2_/FiO_2_ ratio thresholds established in invasively ventilated patients with ARDS cannot be directly extrapolated to this population. Nevertheless, the oxygenation indices retain clinical relevance under HFNO, as lower values remain associated with worse outcomes, reflecting more severe hypoxemia and disease burden. When ABGs are sparse, SpO_2_/FiO_2_ trends and composite indices that incorporate respiratory rate (ROX index=[SpO_2_/FiO_2_]/respiratory rate) may be useful***,*** but absolute cutoffs beyond ARDS (e.g., SpO_2_/FiO_2_ 235/315) translate poorly; device- and patient-specific FiO₂ variability widens error bands.

### Noninvasive ventilation

NIV introduces additional uncertainty concerning the FiO₂ delivery due to potential leaks and air entrainment at the interface. These factors can significantly lower the delivered FiO_2_ and thereby distorting the oxygenation ratios. Both bench and clinical studies have demonstrated that the presence of leaks can result in a considerable reduction in FiO_2_ and risk overestimation of oxygenation status.^[^^55^^,^^60^^]^ In the post hoc analysis of the LUNG SAFE study, the severity assessment by PaO_2_/FiO_2_ ratio on NIV was associated with greater intensity of ventilatory support and higher ICU mortality compared to patients on invasive ventilation, particularly among those with a PaO_2_/FiO_2_ <150 mmHg.^[^^61^^]^ Nevertheless, SpO_2_/FiO_2_-based rules yield important prognostic information in patients with mixed-etiology AHRF on NIV, with a low SpO_2_/FiO_2_ ratio is indicative of higher likelihood of NIV failure.^[^^62^^]^

## Limitations and Challenges with Oxygenation Ratios

The ongoing pursuit of an ideal oxygenation matrix in the context of AHRF and ARDS remains a critical area of research. Each index has unique advantages and limitations, and its application may be influenced not only by its inherent limitations but also by a multitude of patient and institutional factors. Consequently, it is imperative to judiciously evaluate these factors when designing or interpreting the results of any clinical trial or translating the evidence at the bedside for decision–making.

Traditionally, the PaO_2_/FiO_2_ ratio has been used for the diagnosis, management, and prognostication of ARDS.^[^^2^^,^^11^^]^ For most clinical situations, the PaO_2_/FiO_2_ ratio remains the gold standard in the management of AHRF. The PaO_2_/FiO_2_ ratio cutoff for categorizing ARDS was, however, challenged by many dataset analyses, especially the threshold of 300 mmHg.^[^^63^^,^^64^^]^ A recent updated meta-analysis of 28 clinical trials involving 38,270 patients reported a lower accuracy of the PaO_2_/FiO_2_ ratio in predicting mortality for patients with ARDS and recommended that the ratio may not be suitable for use as a sole clinical prognostic tool. They reported an AUROC of 0.60 (95%CI: 0.58 to 0.64) for PaO_2_/FiO_2_ of 100 mmHg, with a pooled sensitivity and specificity of 44.8% (95%CI: 38.1 to 51.7%), and 70.6% (95%CI: 65.9 to 74.9%), respectively. In contrast, for a PaO_2_/FiO_2_ ratio of 200 mmHg, the AUROC was 0.64 (95%CI: 0.60 to 0.69), with pooled sensitivity and specificity of 83.9% (95%CI: 78.9 to 87.8%) and 26.1% (95%CI: 20.8 to 32.1%), respectively.^[^^65^^]^

The PaO_2_/FiO_2_ ratio is affected by various factors such as switching from non-invasive to invasive mechanical ventilation and ventilatory settings, for example, PEEP, FiO_2_, mean airway pressure, and minute ventilation. An important limitation of the PaO_2_/FiO_2_ ratio is its nonlinear relationship with FiO_2_, whereby changes in FiO_2_ can lead to disproportionate and sometimes misleading changes in the PaO_2_/FiO_2_ ratio, independent of true alterations in pulmonary gas exchange. This phenomenon reflects the complex interplay between ventilation–perfusion mismatch, shunt fraction, and oxygen–hemoglobin dissociation curve.^[^^66^^]^ As a result, identical PaO_2_/FiO_2_ ratios may represent different degrees of lung injury at varying FiO_2_ levels, limiting their reliability as a standalone marker of disease severity. The nonlinear PaO_2_/FiO_2_–FiO_2_ relationship underscores the need to interpret the PaO_2_/FiO_2_ ratio within the broader clinical and ventilatory context. The dependence on FiO_2_ may also diminish the utility of these indices, as FiO_2_ can be influenced by various factors, such as mode of oxygen delivery (nasal prongs, face mask, HFNO, NIV, or invasive mechanical ventilation) and ventilatory settings.^[^^49^^]^

Moreover, PaO_2_/FiO_2_ requires arterial sampling, which is invasive, painful, and may not be readily available, especially in resource-limited settings. It also requires technical expertise, and its processing requires special apparatus, which is expensive, needs maintenance, and is not widely available. It may not be possible to store and transport ABG samples, as this may affect PaO_2_ readings.^[^^2^^]^

The SpO_2_/FiO_2_ ratio has been suggested as a potential surrogate and may be particularly useful in situations where ABG sampling is unavailable or not feasible.^[^^11^^,^^67^^]^ The seminal ARDS work that popularized SpO_2_/FiO_2_ cutoffs cautioned about its use at high saturations.^[^^13^^]^ The correlation between SpO_2_/FiO_2_ and PaO_2_/FiO_2_ ratios has been evaluated in several recent studies.^[^[13], [28], [29], [33], [68], [69]^]^ Despite numerous studies, the correlation between PaO_2_/FiO_2_ and SpO_2_/FiO_2_ ratios, is moderate to strong.^[^[26], [32], [34], [70]^]^ Moreover, the oxyhemoglobin curve plateaus above SpO_2_ >96%, and imputation of PaO_2_/FiO_2_ from SpO_2_/FiO_2_ may become inaccurate.^[^^71^^]^ Therefore, different SpO_2_/FiO_2_ thresholds have been identified across studies, and different equations have been proposed for imputing PaO_2_/FiO_2_ from SpO_2_/FiO_2_.^[^^26^^,^^36^^,^^72^^]^ The challenges exist in using SpO_2_/FiO_2_ and PaO_2_/FiO_2_ interchangeably, especially at higher FiO_2_, and in monitoring the severity and progression of ARDS.^[^^68^^,^^70^^,^^73^^]^ Imprecision of the SpO_2_ measurement and these equations may lead to misclassification of ARDS based on the SpO_2_/FiO_2_ ratio, which could be as high as 33% of patients. Moreover, the accuracy does not improve with dynamic monitoring of the SpO_2_/FiO_2_ ratio.^[^^72^^]^ Therefore, panelists from a recent international Delphi study on defining and subphenotyping ARDS expressed reservations regarding the routine implementation of the SpO_2_/FiO_2_ ratio, and indicated the need for further validation before its acceptance in future ARDS definitions.^[^^73^^]^

Furthermore, several other factors may also limit the clinical applicability of the SpO_2_/FiO_2_ ratio.^[^^2^^,^^49^^]^ The SpO_2_/FiO_2_ ratio is dependent on pulse oximetry readings, which can be influenced by various factors such as anemia, skin color, carbon monoxide intoxication, methemoglobinemia, certain dyes and pigments (e.g., nail polish), hyperbilirubinemia, hypoperfusion, high venous pressure, hypoxemia, and hyperoxia.^[^^49^^]^ Moreover, pulse oximetry was associated with greater occult hypoxemia and can artifactually inflate the SpO_2_/FiO_2_ ratio, particularly in darker-skinned patients.^[^^74^^]^ The hidden hypoxia was particularly higher among Black individuals and those of African American ethnic background.^[^^75^^]^ Moreover, this overestimation of oxygen saturation translates into health disparities with delayed treatment and higher readmission to the hospital among patients with dark-skin pigmentation.^[^^76^^]^ Even excessive patient movements can impact the accuracy of pulse oximetry readings. Given the common presence of these factors in critically ill patients, the validity of pulse oximetry readings may be compromised.

### Beyond oxygenation ratios

Although oxygenation indices are surrogates of lung injury severity and are valuable in clinical decision-making, their interpretation is influenced by the complex interplay of ventilation–perfusion mismatch, shunt fraction, and the level and type of respiratory support provided. As a result, patients with markedly different pathophysiological profiles may have similar PaO_2_/FiO_2_ ratios, thereby complicating risk stratification and limiting treatment precision.^[^^66^^]^ Despite the ease of use of oxygenation ratios, their limitations are becoming increasingly recognized. Several additional or complementary metrics have been made available to support a more comprehensive evaluation of respiratory function, potentially better capturing the interactions among oxygenation, lung mechanics, and ventilator settings. We have categorized these indices into: those readily available at bedside and do not require advanced ventilatory maneuvers; those requiring specific ventilatory maneuvers/calculations, and those requiring additional devices.

## Indices not Requiring Specific Ventilator Maneuvers

PaO_2_/FiO_2_ ratio does not account for airway pressures, and thus, investigators included PEEP as the (PaO_2_ ×10) / (FiO_2_ × PEEP) ratio,^[^^77^^]^ or mean airway pressure as an oxygenation factor,^[^^78^^]^ as more integrative metrics. Both indices have been shown to be superior in predicting severity and patient outcomes.

Oxygenation factor=PaO_2_/FiO_2_/Paw, where Paw is the mean airway pressure.

Similarly, oxygenation index (Paw × 100 × FiO_2_/PaO_2_) and surrogate oxygen saturation index (Paw × 100 × FiO_2_/SpO_2_) incorporating mean airway pressure have been found to be superior predictors of outcomes than traditional oxygenation ratios.^[^^79^^,^^80^^]^

The dynamic trajectory of the PaO_2_/FiO_2_ ratio or reclassification of severity after 24–48 h has also been found to be a better predictor of prognosis, response to interventions such as PEEP, and patient outcomes than static measurements.^[^[81], [82], [83]^]^

## Indices Requiring Specific Ventilatory Maneuvers or Calculations

Lung compliance and elastance, calculated from plateau pressure and tidal volume, reflect the respiratory system’s mechanical properties. Lower compliance and higher elastance frequently suggest more severe lung parenchymal involvement or atelectasis, and are independently linked with death.^[^^84^^]^ Combining oxygenation indices and compliance (for example, the “oxygenation-compliance index”) may aid in the identification of patients with recruitable lung tissue or different physiological phenotypes.^[^^85^^,^^86^^]^ For example, two patients with equal PaO_2_/FiO_2_ ratios may differ significantly in compliance—one with recruitable atelectasis, the other with fixed fibrotic change—implying very different responses to PEEP or prone positioning (Figure 1).

**Figure 1:**
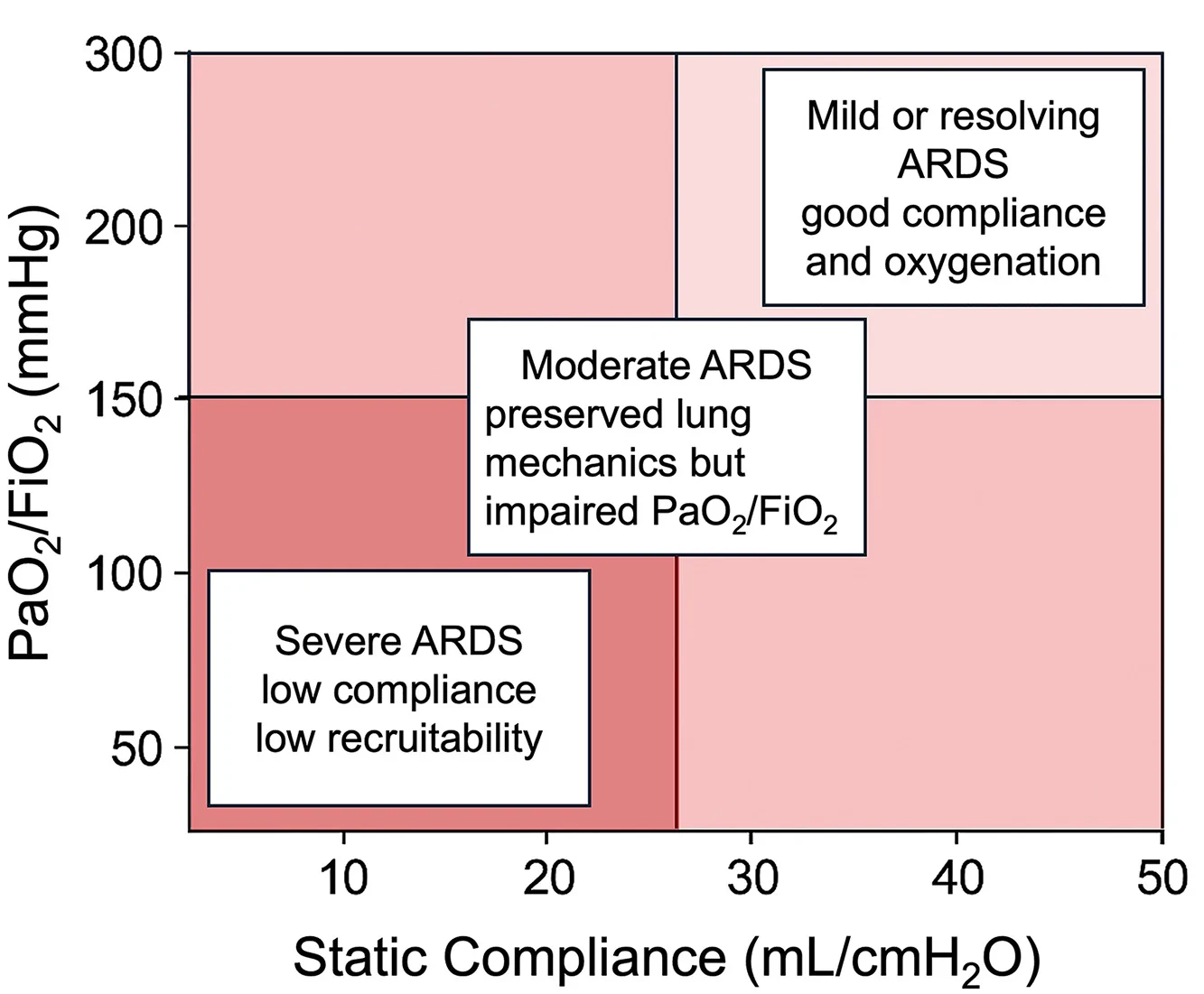
Relationship between oxygenation indices and respiratory system compliance to define physiological phenotypes in ARDS. The figure illustrates the relationship between oxygenation and static compliance, highlighting that patients with similar PaO_2_/FiO_2_ ratios may have markedly different respiratory mechanics, reflecting distinct underlying pathophysiology. Three representative phenotypes are shown: PaO_2_/FiO_2_ with preserved compliance (recruitable atelectasis), suggesting potentially recruitable lung units despite impaired oxygenation; low PaO_2_/FiO_2_ with low compliance (fibrotic or nonrecruitable lung), consistent with severe, poorly recruitable lung injury; and preserved PaO_2_/FiO_2_ with high compliance, representing mild or resolving disease. Integrating oxygenation and compliance offers a more comprehensive, physiology-based assessment of ARDS, enabling better phenotyping and more individualized ventilatory management than either parameter alone. ARDS, Acute respiratory distress syndrome; PaO_2_/FiO_2_, Partial pressure of oxygen to fraction of inspired oxygen.

Similarly, the dead space fraction (Vd/Vt) measures ventilation efficiency and pulmonary perfusion. Elevated dead space can be caused by alveolar overdistension, microvascular obstruction, or thrombotic complications, none of which are reflected in oxygenation measurements. Increased Vd/Vt has been repeatedly linked to poor outcomes in ARDS and may act as a surrogate for pulmonary vascular dysfunction, supplementing oxygenation-based measurements.^[^^87^^,^^88^^]^ Surrogates, such as the ventilatory ratio (VR)^[^^89^^]^ or the PaCO_2_/end-tidal carbon dioxide (etCO_2_) gradient, can be used at the bedside when direct measures of dead space are unavailable.^[^[90], [91], [92]^]^

The shunt fraction is the proportion of cardiac output that perfuses nonventilated regions and directly quantifies the severity of intrapulmonary shunt. Although perfect estimation necessitates invasive sampling or numerous gas measurements, simple calculations using alveolar gas equations can approximate the shunt fraction. Integrating shunt, dead space, and compliance data may assist in distinguishing “low-compliance, high-shunt” from “high-compliance, high-dead-space” phenotypes, which respond differently to PEEP, recruitment, or prone positioning.^[^^92^^,^^93^^]^

Recent research has examined composite measures that combine oxygenation and mechanics. The “oxygenation–ventilation index,” “mechanical power,” and “stress index” take into account pressure–volume relationships, respiratory rate, and driving pressure to measure both the efficiency and potential harm caused by mechanical ventilation. These multidimensional metrics better reflect the interactions among gas exchange, mechanics, and ventilator settings, improving predictive power and therapy personalization.^[^^94^^,^^95^^]^

## Indices Requiring Additional Devices

Beyond traditional physiological parameters, innovative monitoring technologies provide novel insights into regional and dynamic variation. Electrical impedance tomography (EIT) provides for continuous bedside monitoring of regional ventilation and, with newer algorithms, perfusion distribution. EIT can assess regional compliance and detect overdistension or collapse during PEEP titration, bridging the gap between global indices and regional physiology. Lung ultrasound and quantitative CT help to characterize aeration and recruitability, while newer techniques such as functional lung MRI or dual-energy CT allow for noninvasive measurement of ventilation–perfusion mismatch and shunt percentage. Integrating these imaging modalities with standard measurements has the potential to reinvent severity classification, shifting away from static thresholds (such as PaO_2_/FiO_2_ cutoffs) toward physiologically grounded multidimensional metrics.^[^^4^^]^

Despite these encouraging developments, major issues remain. Many of these measurements necessitate specialized equipment, are operator-dependent, or lack standardized thresholds. Furthermore, their interpretation is complicated by ventilatory settings, hemodynamics, and metabolic state. To translate these intricate factors into effective bedside algorithms, it is essential to conduct large, prospective cohort studies that are integrated with patient outcomes. Notably, many of these indices either incorporate the PaO_2_/FiO_2_ ratio or complement these indices to support more precise, physiology-guided management. Therefore, the purpose is not to replace the PaO_2_/FiO_2_ ratio, but to contextualize it within a broader framework that includes both gas-exchange efficiency and respiratory system mechanics.

## Research Priorities and the Way Forward

The limitations of current oxygenation metrics demand a systematic research agenda to develop more nuanced, context-specific approaches to respiratory assessment and care. Future investigations must move beyond simple oxygenation ratios validation toward a comprehensive evaluation of how oxygenation measures should be integrated into modern clinical practice.^[^^96^^]^

The thresholds for PaO_2_/FiO_2_ ratio were established decades ago from relatively small datasets that do not reflect contemporary patient populations, ventilation strategies, or outcomes. Large-scale analyses of modern ICU databases could identify cutoffs that better align with current mortality patterns, length of stay, and resource utilization. Machine learning techniques, applied across diverse healthcare systems, might reveal that optimal thresholds vary substantially by age, comorbidity burden, or underlying pathophysiology.^[^^97^^,^^98^^]^ Importantly, such efforts should prioritize clinically meaningful outcomes over historical precedent, recognizing that different thresholds may more accurately predict treatment response, recovery trajectory, or the need for escalation of care.

Equally important is the recognition that universal cutoffs may be fundamentally flawed. HFNO, for example, produces unique physiological conditions in which traditional PaO_2_/FiO_2_ or SpO_2_/FiO_2_ thresholds poorly predict treatment failure or the need for intubation. Systematic studies are urgently required to evaluate oxygenation metrics across the full spectrum of respiratory support modalities, from conventional oxygen therapy and NIV to HFNO and invasive ventilation. These investigations should aim to establish context-specific thresholds that reflect the distinct physiological states created by each intervention. Patient-specific factors must also be considered. The optimal oxygenation target for an elderly patient with multiple comorbidities may differ substantially from that for a young, otherwise healthy patient with isolated respiratory failure (Figure 2).

**Figure 2:**
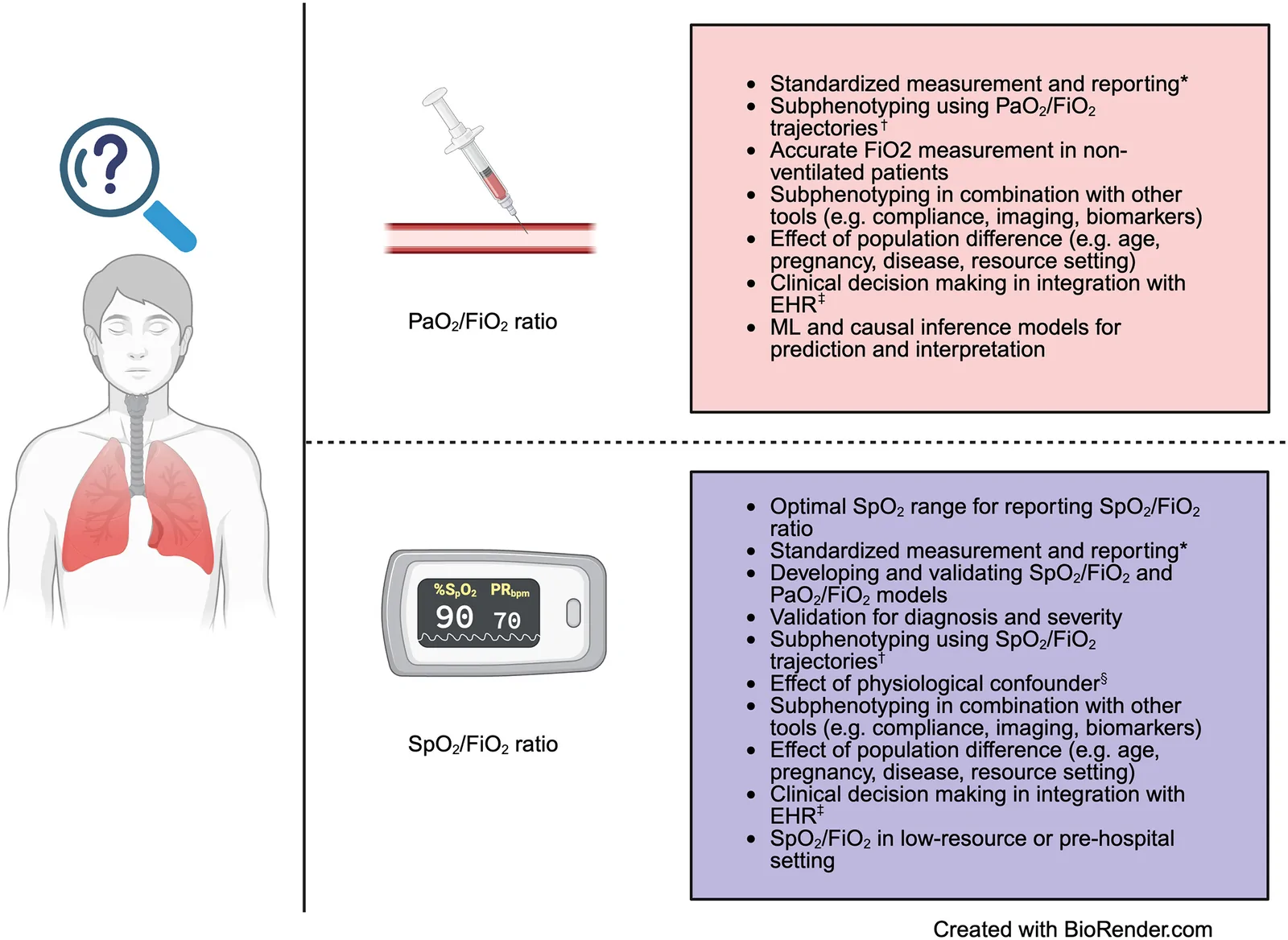
Research priorities for oxygenation metrics. EHR, Electronic health record; ML, Machine learning; PEEP, Positive end-expiratory pressure; PaO_2_/FiO_2_, Partial pressure of oxygen to fraction of inspired oxygen; SpO_2_/FiO_2_, Capillary oxygen saturation to fraction of inspired oxygen; *Standardized measurement includes ventilator settings, FiO_2_, PEEP, flow rate, patient position, time relative to intervention, vasopressors, and skin pigmentation. ^†^Subphenotyping using longitudinal trajectories of PaO_2_/FiO_2_ or SpO_2_/FiO_2_ ratios over static measurement. ^‡^Integration, automatic extraction, and validation of PaO_2_/FiO_2_ and SpO_2_/FiO_2_ from EHRs for alerts, dashboards, and research phenotyping. ^§^Physiological confounders include effect of pH, arterial partial pressure of carbon dioxide, temperature, hemoglobin, 2,3-diphosphoglycerate (DPG), dyshemoglobinemias and cardiac output.

Beyond refining thresholds, future research should move away from single-parameter assessments. Composite indices that capture the complexity of respiratory failure hold greater promise. Metrics combining oxygenation with measures of lung mechanics, such as PEEP, mean airway pressure, lung compliance and driving pressure, gas exchange efficiency, including dead space fraction, hemodynamic parameters, and inflammatory markers, could yield a more accurate and clinically useful assessment. Integration of routinely available ventilator–derived data, such as pressure-volume loops, flow patterns, or indices of work of breathing, with oxygenation measures could enhance bedside evaluation without adding invasive monitoring.^[^^99^^]^ Emerging technologies, including lung ultrasound and EIT, further expand opportunities for composite metric development. Studies linking these modalities with traditional oxygenation indices may provide more reliable insight into regional lung function, recruitment potential, and respiratory reserve.

Crucially, research priorities must extend beyond the development of new metrics to their implementation in clinical practice. Understanding how different oxygenation assessment strategies affect clinician decisions, workflow efficiency, and patient comfort represents a major knowledge gap. Studies assessing the cognitive load imposed by various monitoring approaches and their influence on provider satisfaction are necessary for successful adoption.

The ultimate future of oxygenation assessment may lie not in refining individual metrics but in developing intelligent, context-aware systems that integrate multiple data streams to provide personalized respiratory evaluation. Such systems should account for patient-specific factors, treatment context, and resource constraints while maintaining the simplicity required for widespread clinical use.

In summary, the path forward involves a shift from reliance on simple, universal ratios toward sophisticated, adaptable approaches that reflect the complexity of respiratory failure. Progress will depend on combining modern analytics, advanced monitoring, and pragmatic implementation research to ensure that innovations translate into real-world benefit. The ultimate goal is to have oxygenation assessment strategies that improve patient outcomes while reducing the burden on healthcare systems in an era of increasingly personalized, resource–conscious medicine.

## Conclusions

Oxygenation ratios provide simple and rapid estimates of lung injury, enabling bedside assessment of ARDS severity and prognostication. They are instrumental in screening, triage, and trend monitoring for standardized interventions. However, both metrics, PaO_2_/FiO_2_ and SpO_2_/FiO_2_ ratios, can be misleading and are highly influenced by the ventilatory settings such as PEEP, mean airway pressure, and FiO_2_. The SpO_2_/FiO_2_ ratio is a promising alternative to the PaO_2_/FiO_2_ ratio, especially in resource–limited settings, but physiologic nonlinearity, FiO₂ variability, and oximetry bias limit precision around decision thresholds. There is a need for research on the validation of oxygenation ratios in diverse clinical settings, modes of oxygen delivery, and integration with other oxygenation indices, including pulmonary mechanics. Although oxygenation ratios are valuable screening and monitoring tools, they should be interpreted within the broader clinical and physiological context rather than as standalone markers of gas exchange and disease severity.

## CRediT authorship contribution statement

**Prashant Nasa:** Writing – review & editing, Writing – original draft, Data curation, Conceptualization. **Denise Battaglini:** Writing – review & editing, Writing – original draft, Conceptualization. **Deven Juneja:** Writing – review & editing, Writing – original draft, Conceptualization. **Marcus J. Schultz:** Writing – review & editing, Writing – original draft, Supervision, Conceptualization. **Jozef Kesecioglu:** Writing – review & editing, Writing – original draft, Supervision, Methodology, Conceptualization.
